# Primary Ciliary Dyskinesia with Secondary *Pseudomonas aeruginosa* Infection in a Dog: Endoscopic and Diagnostic Findings †

**DOI:** 10.3390/vetsci13101114

**Published:** 2026-10-19

**Authors:** Giulia Maggi, Francesca Pirgher, Davide De Lorenzi, Luca Aresu, Maria Chiara Marchesi

**Affiliations:** 1 Department of Veterinary Medicine, University of Perugia, Via San Costanzo 4, 06126 Perugia, Italy; francesca.pirgher@gmail.com (F.P.); maria.marchesi@unipg.it (M.C.M.); 2 San Marco Veterinary Clinic and Laboratory, Via dell’Industria 3, 35030 Veggiano, Italy; 3 Department of Veterinary Sciences, University of Turin, Largo Braccini 2, 10095 Grugliasco, Italy; luca.aresu@unito.it

**Keywords:** primary ciliary dyskinesia, endoscopy, respiratory infection, dogs

## Abstract

A 7-month-old, 3.6-kg, intact male Dachshund was presented with a lifelong history of chronic respiratory signs. Multiple courses of antimicrobial therapy resulted in partial or complete clinical improvement, followed by recurrence after treatment discontinuation. To investigate the underlying cause of the persistent respiratory disease, rhino-tracheobronchoscopy was performed. Bronchoalveolar lavage (BAL) fluid and respiratory mucosal biopsy specimens were collected for cytological, histopathological, and ultrastructural evaluation. Cytological examination of BAL fluid revealed septic neutrophilic inflammation, and bacterial culture yielded *P. aeruginosa.* Transmission electron microscopy (TEM) identified ultrastructural abnormalities of the ciliary axoneme in 50% of the examined respiratory cilia. Based on these findings, primary ciliary dyskinesia (PCD) was diagnosed. PCD is a rare congenital disorder characterized by structural and functional abnormalities of motile cilia, resulting in impaired mucociliary clearance and recurrent respiratory infections. This case highlights the diagnostic value of a detailed clinical history, endoscopic examination, and TEM in dogs with suspected PCD.

## Simple Summary

A young dog was presented with a lifelong history of chronic respiratory signs that partially improved with antibiotic treatment but repeatedly returned after therapy was stopped. To investigate the underlying cause, endoscopic examination of the respiratory tract was performed, allowing the collection of several biological samples for further analysis. Ultrastructural examination and microbiological testing led to a diagnosis of primary ciliary dyskinesia associated with *Pseudomonas aeruginosa* infection. Primary ciliary dyskinesia is a rare congenital disorder characterized by abnormalities in the structure and movement of cilia, including those lining the respiratory tract. These cilia play an important role in protecting the airways by removing mucus, microorganisms, and other inhaled particles. When their function is impaired, affected animals may develop recurrent or persistent respiratory infections. This case highlights the importance of clinical history, endoscopic examination and transmission electron microscopy in the diagnosis of primary ciliary dyskinesia.

## 1. Introduction

Primary ciliary dyskinesia (PCD) is a rare congenital ciliopathy characterized by ultrastructural abnormalities of the cilia. The clinical presentation depends on the location of the affected cilia and the presence of concurrent congenital abnormalities, which may include situs inversus, congenital cardiac defects, and other developmental anomalies [1]. Involvement of the respiratory cilia impairs mucociliary clearance and predisposes affected individuals to recurrent respiratory infections, leading to chronic rhinosinusitis, otitis media, bronchitis, bronchopneumonia, and, in advanced cases, bronchiectasis [1]. Other reported clinical manifestations include male and female infertility, hydrocephalus, and, less commonly, renal tubular dilatation and renal fibrosis. Due to its congenital origin, PCD primarily affects young animals, although clinical signs may not become apparent until several months of age in some individuals [2]. Extensive scientific literature in both human and veterinary medicine supports a genetic basis for PCD, most commonly involving autosomal recessive inheritance [3,4,5].

The diagnosis of PCD relies on the identification of characteristic ultrastructural abnormalities of the cilia. To date, PCD has been classified into six distinct ultrastructural types based on the specific morphological defect observed. Transmission electron microscopy (TEM) remains the gold standard for both diagnosis and classification, as it allows detailed evaluation of ciliary ultrastructure and quantification of the proportion of abnormal cilia [6]. However, chronic inflammation of the respiratory tract may induce secondary ultrastructural abnormalities of the cilia. These changes may include the formation of compound cilia containing multiple axonemes, which may exhibit incomplete assembly and/or abnormal orientation, as well as alterations of the ciliary membrane and cytoplasmic components. These features differ from the characteristic ultrastructural defects observed in PCD [6,7]. Distinguishing primary from secondary abnormalities may therefore be challenging, particularly in animals with chronic respiratory disease. Furthermore, a subset of patients with PCD may exhibit normal ciliary ultrastructure on TEM despite impaired mucociliary clearance. In these cases, assessment of ciliary function by in vivo or in vitro functional testing may aid diagnosis [2]; however, these techniques are rarely available in veterinary clinical practice.

Therefore, recognition of the characteristic clinical presentation and appropriate diagnostic findings is essential for achieving an accurate diagnosis. The present case report describes the clinical presentation, diagnostic work-up, and endoscopic findings of a dog with PCD presenting with chronic cough, sneezing, and nasal discharge since birth, highlighting the pivotal role of endoscopic evaluation and TEM in the diagnosis of this uncommon disease.

## 2. Case Presentation

**Figure 1:**
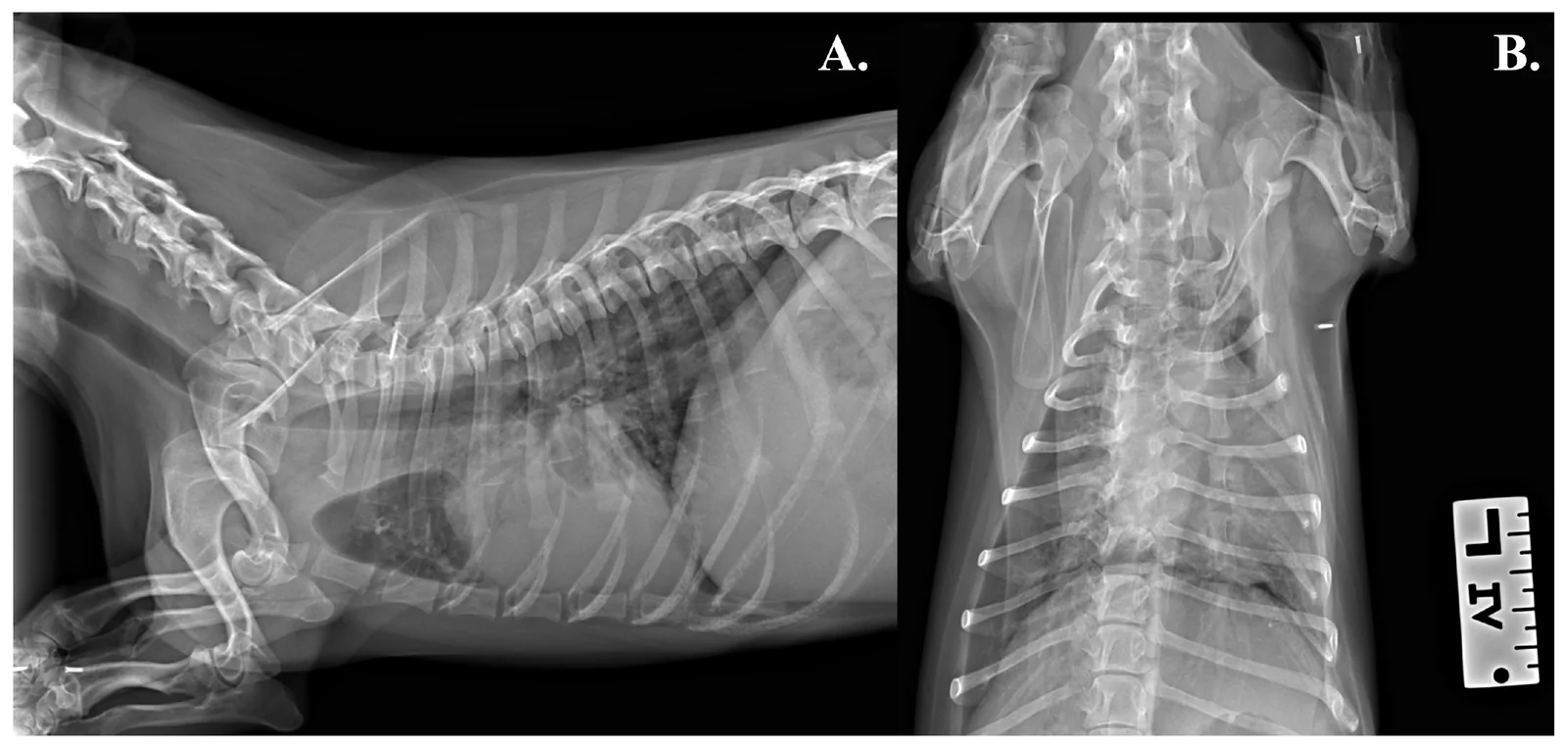
Radiographs of the neck and thorax. (**A**) Latero-lateral radiographic projection of the neck and thorax, showing increased opacity of the left caudal lung lobe with associated volume loss. (**B**) A ventro-dorsal projection of the thorax showing increased consolidation of the left caudal lung lobe consistent with atelectasis.

A 7-month-old, 3.6-kg, intact male Dachshund living indoors was referred for a second opinion because of chronic respiratory signs, including cough, sneezing, and nasal discharge, which had been present since birth. Similar respiratory signs had also been reported in a littermate. Previous bacterial cultures of nasal swab samples collected from both animals by the referring veterinarian yielded *Acinetobacter radioresistens*. Furthermore, repeated courses of antimicrobial therapy, including amoxicillin–clavulanic acid, cephalosporins, and doxycycline, resulted in transient clinical improvement, with recurrence of respiratory signs after treatment cessation. Physical examination revealed macrocrania, opaque coat, bilateral mucous nasal discharge, diffuse fine crackles on thoracic auscultation, and expiratory dyspnea. A complete blood count (CBC) and serum biochemical analysis were performed, revealing a mild leukocytosis, primarily due to neutrophilia. Latero-lateral (LL) and ventro-dorsal (VD) radiographs of the neck and thorax were obtained, showing consolidation of the left caudal lung lobe consistent with atelectasis (Figure 1).

**Figure 2:**
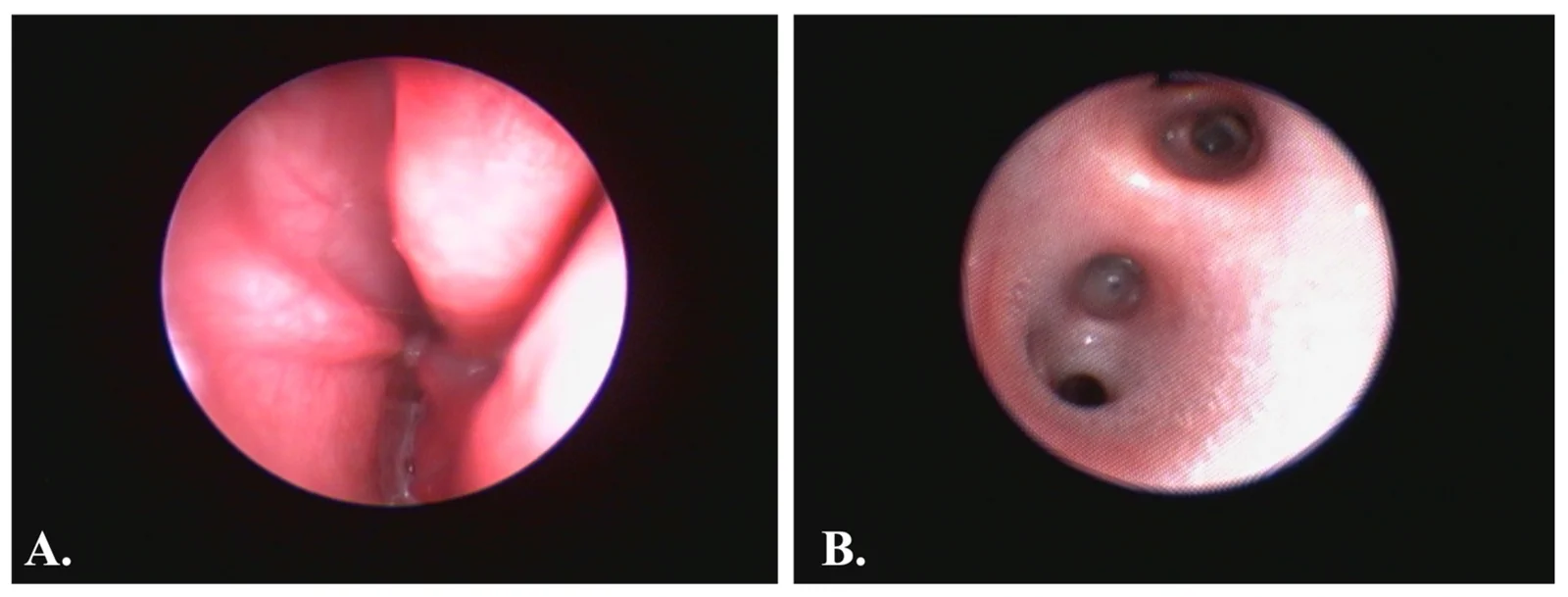
Endoscopic findings. (**A**) Rhinoscopic image showing abundant mucus within the nasal cavity. (**B**) Bronchoscopic image showing abundant, foamy mucus within the lumen of the left caudal bronchus (LB2).

Serum concentrations of total immunoglobulins (Ig)G and IgM were within normal limits. A Baermann test was performed to investigate pulmonary nematode infection and yielded a negative result. Hematological and radiographic findings were consistent with pneumonia. Therefore, the dog underwent rhino-tracheobronchoscopic examination, which revealed abundant, dense mucus within both nasal cavities, without endoscopic evidence of nasal mucosal hyperemia or edema, as well as within the segmental and subsegmental branches of the left caudal bronchus (LB2) (Figure 2).

Under endoscopic guidance, a bronchoalveolar lavage (BAL) was performed in LB2 for cytological, bacteriological analysis and polymerase chain reaction (PCR) research of *Pneumocystis carinii*. Consequently, bronchial, tracheal, and nasal biopsy specimens were obtained concurrently for histopathological and ultrastructural evaluation and were fixed in formalin and glutaraldehyde, respectively. Specifically, two specimens were obtained from the middle meatus of each nasal cavity, two from the cervical trachea, one from the carina, and two from each of the right and left caudal bronchi. In addition, a nasal swab was obtained for bacteriological culture. Cytological examination of BAL fluid revealed septic neutrophilic inflammation, whereas histological examination of the trachea, bronchi and nasal mucosa was unremarkable. No evidence of *P. carinii* infection was detected by PCR. Subsequently, both samples underwent bacteriological examination, including inoculation onto selective and differential culture media, followed by identification of the bacterial isolates using matrix-assisted laser desorption/ionization time-of-flight mass spectrometry (MALDI-TOF MS). *P. aeruginosa* was identified in both samples.

**Figure 3:**
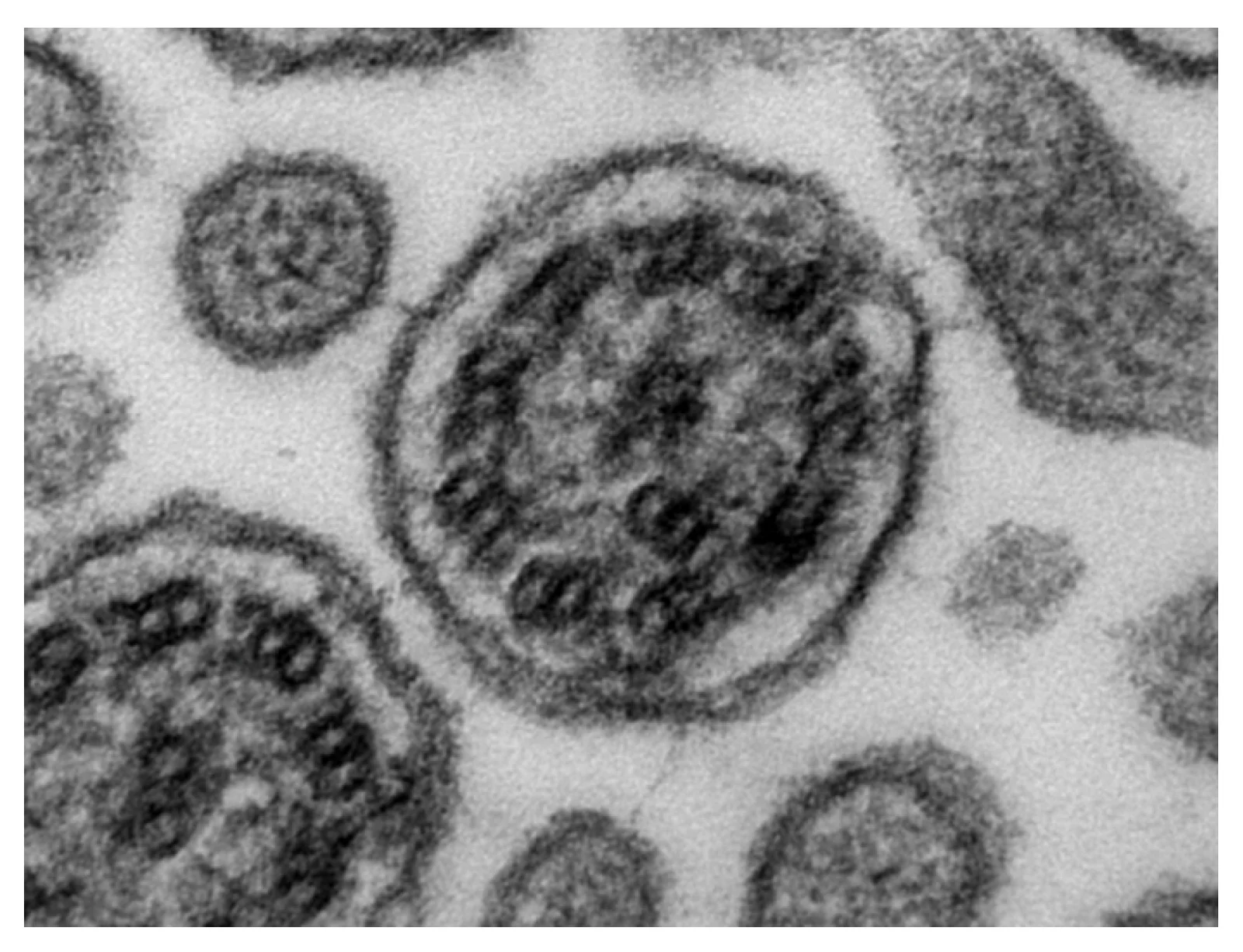
Transmission electron microscopy (TEM) of examined cilia. TEM cross-sections of respiratory cilia showing abnormalities of the radial spokes, with variably expressed defects ranging from their complete absence to the lack of the central sheath.

TEM revealed ultrastructural alterations in the axonemal architecture of 50% (15/30) of the respiratory cilia examined in cross-section, obtained from a 5-mm respiratory mucosal biopsy specimen. Morphological alterations included abnormalities of the radial spokes, with variably expressed defects ranging from their complete absence to the lack of the central sheath. These changes resulted in the loss of the typical ‘9 + 2’ axonemal organization and axial symmetry, with alterations ranging from disorganization of the central region to irregular configurations of the central complex and abnormal spatial relationships among the peripheral doublets (Figure 3). The ultrastructural findings observed, ranging from radial spoke defects to alterations of the central complex with loss of axonemal symmetry, were overall consistent with a type II-PCD.

Overall, these findings were consistent with PCD complicated by secondary infection with *P. aeruginosa*.

To further assess ciliary function, we also attempted to collect a semen sample for functional and morphological evaluation of the spermatozoa. However, an adequate sample could not be obtained, as the dog was only 6 months old and had not yet reached sexual maturity. Following diagnosis, genetic testing for the breed-associated CCDC39 variant c.286C>T was performed in both the dog and its sibling, which exhibited similar clinical signs. Genotyping was carried out using an allele-specific real-time PCR assay. The c.286C>T variant was not detected in either dog, and both animals were homozygous for the wild-type allele.

The antimicrobial susceptibility profile of the *P. aeruginosa* isolate was assessed using the Kirby–Bauer disk diffusion method, in accordance with the Clinical and Laboratory Standards Institute (CLSI) guidelines. The results of the antimicrobial susceptibility testing are reported in Table 1.

This treatment was combined with anti-inflammatory therapy consisting of prednisolone, administered orally at a starting dose of 0.5 mg/kg every 12 h for 15 days, followed by a gradual dose tapering. Mucus fluidification was supported with aerosol therapy using NaCl and oral N-acetylcysteine. After discontinuation of the antibiotic and anti-inflammatory therapy, the dog exhibited regular breathing; thoracic auscultation revealed only reinforced vesicular sounds, with sporadic cough and persistent nasal discharge. Follow-up CBC and thoracic radiographs performed after therapy revealed no abnormalities. Fluidification strategies were maintained to prevent recurrence of infections. Following the initial treatment, the dog remained largely asymptomatic, with only mild mucoid nasal discharge persisting for several months. However, after five months of clinical improvement maintained with aerosol therapy alone, the dog experienced a first clinical relapse, which was treated with the same therapeutic protocol for 15 days. Shortly after completion of treatment, the dog developed a second, more severe relapse characterized by pneumonia and marked respiratory distress, and died a few days later.

## 3. Discussion

This case report describes a 7-month-old Dachshund diagnosed with PCD based on a history of persistent respiratory clinical signs, including chronic cough, sneezing, and nasal discharge. The case highlights the diagnostic value of a thorough medical history combined with clinical and pathological findings including TEM performed on samples collected during endoscopy, in dogs with suspected PCD.

The careful evaluation of the dog’s medical history and signalment played a crucial role in the diagnostic process. The presence of clinical signs since birth, together with a similar clinical history in its littermate, raised suspicion of an inherited and/or congenital disorder. The chronicity of the respiratory signs, together with the incomplete response to antimicrobial treatment and subsequent relapses, further raised suspicion of an underlying disorder associated with recurrent respiratory infections. Given the young age of the dog and the presence of recurrent respiratory infections, a primary immunodeficiency disorder was initially suspected. A rare form of immunodeficiency associated with chronic respiratory clinical signs and hypogammaglobulinemia, involving IgA, IgG, and IgM, has been described in miniature Dachshunds [8,9]. However, in the present case, serum concentrations of IgG and IgM were within the reference intervals, providing no evidence of hypogammaglobulinemia or an apparent humoral immunodeficiency. Despite normal serum immunoglobulin concentrations, opportunistic infections were considered, also given the recurrent respiratory signs following discontinuation of antibiotic therapy.

Another form of impairment of local respiratory defense is represented by defective mucociliary clearance, which occurs in PCD [10]. The respiratory epithelium is characterized by a pseudostratified columnar epithelium composed of ciliated epithelial cells. The coordinated movement of the cilia, which are located at the apical surface of these cells, represents a fundamental defense mechanism against numerous pathogens throughout the respiratory tract. Impairment of mucociliary clearance compromises this protective mechanism, increasing susceptibility to respiratory infections and contributing to chronic inflammatory changes affecting both the upper and lower respiratory tracts [6]. Physical examination of this case revealed bilateral mucoid nasal discharge, diffuse fine crackles on thoracic auscultation, and expiratory dyspnea, indicative of involvement of both the upper and lower respiratory tracts. In addition, hematological abnormalities, including neutrophilic leukocytosis, and thoracic radiographic findings were suggestive of pneumonia. Another relevant clinical finding was macrocrania, which raised suspicion of underlying hydrocephalus, a neurological abnormality previously reported in association with PCD [1]. However, this possibility could not be confirmed because magnetic resonance imaging (MRI), which would have been required for definitive diagnosis, was not performed due to the owner’s financial constraints. Although a direct causal relationship between PCD and hydrocephalus has not been established, dysfunction of ependymal cilia has been proposed as a possible pathogenic mechanism, as impaired ciliary motility may interfere with the normal circulation and drainage of cerebrospinal fluid [10,11]. Other congenital abnormalities associated with PCD, such as situs inversus and cardiac abnormalities, were not identified on thoracic radiography or cardiac auscultation, respectively. No evidence of renal dysfunction was detected based on hematological and serum biochemical parameters [1,6].

Due to the persistent and chronic nature of the clinical signs, endoscopic evaluation of the upper and lower airways was performed to investigate the possibility of PCD. The endoscopic examination revealed abundant, tenacious mucus within both nasal cavities and the LB2, findings suggestive of impaired mucociliary clearance. The adjunctive diagnostic value of endoscopy was represented by the possibility to collect biopsies from the nasal, tracheal, and bronchial mucosa. Samples were fixed in formalin for histopathological examination and in glutaraldehyde for ultrastructural evaluation. In fact, TEM represents the gold standard for the diagnosis and ultrastructural classification of PCD [10]. Motile cilia are composed of a microtubule-based structure, the axoneme, consisting of nine peripheral microtubule doublets surrounding a central pair, forming the characteristic “9 + 2” configuration. The peripheral microtubule doublets are connected to the central pair by protein structures termed radial spokes and are interconnected by protein bridges linking adjacent doublets [11,12]. Each microtubule doublet contains inner and outer dynein arms, which generate the mechanical force required for ciliary motility through ATP hydrolysis. At the base of the axoneme, beneath the terminal plate, lies a short cylindrical structure known as the basal body. The basal body shares the basic microtubular organization of the axoneme but consists of nine peripheral microtubule triplets and lacks a central microtubule pair [4,12]. Primary ciliary ultrastructural abnormalities can be classified into different categories according to the affected axonemal component. Type I defects are characterized by abnormalities of the dynein arms. Type II defects involve alterations of the radial spokes. These may manifest either as a complete absence of these structures or as an absence of the central sheath that normally surrounds the central microtubule pair. Type III abnormalities are characterized by transposition of a peripheral microtubule doublet into the central position, typically associated with absence or disruption of the central pair. Type IV defects are characterized by complete absence of the axoneme and basal body. Type V abnormalities include supernumerary microtubule doublets located either within or outside the axonemal structure, whereas type VI defects involve abnormalities of the basal apparatus [6,12]. Disruption of this highly organized architecture can impair the coordination and effectiveness of ciliary beating, resulting in defective mucociliary clearance. In contrast to the intrinsic ultrastructural abnormalities observed in PCD, nonspecific ciliary abnormalities may arise as a consequence of chronic inflammation of the respiratory tract and therefore represent secondary ciliary changes. These abnormalities may include compound cilia containing incomplete and/or misoriented axonemes; cilia with membrane abnormalities, such as membrane loss, vesiculation, or discontinuities; and cilia or axonemal structures located within the cytoplasm. The latter finding may be observed particularly in proximity to the basal body and may therefore represent a sectioning-related or physiological feature rather than a true ultrastructural abnormality. For this reason, ciliary cross-sections obtained too close to the basal body should also be excluded from ultrastructural assessment [6,10,12]. In the present case, TEM revealed ultrastructural abnormalities affecting 50% of the respiratory cilia examined. The observed alterations predominantly involved the radial spokes, supporting their classification as type II ciliary ultrastructural defects and providing further evidence consistent with a diagnosis of PCD. Alterations in the structure and/or function of respiratory cilia impair the mucociliary clearance of infectious agents and particulate matter from the respiratory epithelium and may consequently contribute to chronic respiratory disease. These alterations may arise from a primary, genetically determined defect of ciliary structure and/or function, as observed in PCD, or may develop secondarily to other disease processes. Distinguishing between primary and secondary ciliary dyskinesia (SCD) can be challenging, particularly in the presence of chronic inflammation or infection. In contrast to PCD, SCD refers to acquired and potentially reversible ultrastructural and functional abnormalities of previously normal cilia, including alterations in ciliary orientation and axonemal structure, resulting from injury to the respiratory mucosa [10,12].

Histological evaluation of the tracheal and nasal mucosal samples revealed no significant abnormalities. The absence of inflammatory lesions or other relevant structural alterations in the nasal biopsies further supported the hypothesis of an underlying functional disorder, and may suggest that *P. aeruginosa* was not actively infecting the nasal cavities but rather represented bacterial colonization or contamination. BAL was performed to further characterize the respiratory inflammatory process. The lower respiratory tract showed findings consistent with bacterial bronchopneumonia, as supported by both cytological and bacteriological examinations. Cytological examination revealed septic neutrophilic inflammation, consistent with an active bacterial component. In fact, *P. aeruginosa* was isolated in association with the cytological identification of rod-shaped bacteria, observed both extracellularly in the background and intracellularly within phagocytic neutrophils.

Antimicrobial susceptibility testing confirmed that the bacterial isolate was susceptible to marbofloxacin, which was therefore initiated to treat the respiratory infection. Prednisolone was administered concurrently as adjunctive anti-inflammatory therapy to control the severe chronic airway inflammation associated with the underlying respiratory disease. Following discontinuation of both therapies, the dog showed an improvement in respiratory function, with a regular breathing pattern for 5 months. Thoracic auscultation revealed only increased vesicular sounds, although occasional coughing episodes and persistent nasal discharge remained. Supportive measures aimed at promoting mucus fluidification and airway clearance were continued to reduce the risk of recurrent infections. However, clinical signs recurred on multiple occasions, and each relapse was managed using the same therapeutic regimen until the dog died six months after diagnosis. The long-term prognosis of dogs with PCD remains poorly characterized, and no reliable estimates of life expectancy are currently available. Clinical outcomes appear to vary considerably among affected dogs, likely depending on disease severity, recurrent respiratory infections, and the extent of progressive pulmonary damage [12,13]. In human patients with PCD, *P. aeruginosa* is among the most frequently isolated respiratory pathogens, with reported prevalence ranging from 9% to 47%, and chronic infection has been associated with more extensive structural pulmonary abnormalities on computed tomography (CT) and poorer clinical outcomes [13]. However, information regarding the prevalence and clinical significance of *P. aeruginosa* infection in dogs with PCD is currently lacking. In the present case, CT could not be performed because of financial constraints; therefore, the extent of pulmonary structural damage could not be assessed. It is possible that undetected and severe pulmonary damage contributed to the unfavorable outcome.

Furthermore, CCDC39 has been identified as a PCD-associated gene in both humans and dogs [3,4,5,14]. Studies in canine populations contributed to the identification of CCDC39 as a PCD-related gene and demonstrated that loss-of-function mutations in its human ortholog account for a substantial proportion of PCD cases characterized by axonemal disorganization and abnormal ciliary beating [3,4,5,14]. Functional studies have shown that CCDC39 is localized within the ciliary axoneme and plays a crucial role in the assembly of inner dynein arms and the dynein regulatory complex, both of which are essential for normal ciliary motility [3,4,5,14]. As part of the diagnostic investigation, genetic testing for the CCDC39 mutation (c.286C>T variant) was performed in the affected dog and its sibling, which exhibited similar clinical signs; however, no pathogenic alleles were identified. Other genes, including STK36 and NME5, have also been associated with PCD in dogs [5,14]. Therefore, the absence of a pathogenic CCDC39 c.286C>T variant in the present case does not exclude a genetic etiology, as mutations in other PCD-associated genes may be involved and the genetic basis of canine PCD remains incompletely characterized. These findings emphasize the importance of integrating clinical evaluation, endoscopic examination, and TEM in the diagnostic approach to PCD, while also highlighting the limited diagnostic sensitivity of currently available genetic testing.

The interpretation of the diagnostic findings in this case is subject to several limitations. Ultrastructural analysis was performed on a limited number of cilia and, although morphological abnormalities were identified, functional assessment of ciliary motility was not performed because this diagnostic technique was not readily available in veterinary clinical practice. Advanced diagnostic imaging, including MRI and CT, which could have provided additional information regarding other abnormalities potentially associated with PCD, such as hydrocephalus, as well as a more detailed assessment of pulmonary changes, was not performed because of the owner’s financial constraints. The genetic investigation was limited to the analysis of the c.286C>T variant in the CCDC39 gene and therefore could not exclude other genetic causes of PCD. Finally, a post-mortem examination was not performed following the fatal clinical relapse, precluding definitive pathological confirmation of the underlying cause of death.

## Figures and Tables

**Table 1:** Antimicrobial susceptibility profile of the *P. aeruginosa* isolate. Antimicrobial susceptibility was evaluated using the disk diffusion method. The table reports the antimicrobial agents tested, the corresponding disk content (µg), and the susceptibility category assigned according to the applicable interpretive criteria. S, susceptible; I, intermediate; R, resistant.

**Antimicrobials**	**Inhibition Zone (mm)**	**R**	**I**	**S**
Gentamicin (Gen, 10 μg)	22			x
Marbofloxacine (Mar, 5 μg)	26			x
Enrofloxacin (Enr, 5 μg)	19		x	
Sulfatrimethoprim (Sxt, 25 μg)	0	x		
Doxycicline (Dox, 30 μg)	0	x		
Ceftriazone (Cro, 30 μg)	0	x		
Ceftazidime (Caz, 30 μg)	0	x		

## Data Availability

The original contributions presented in the study are included in the article; further inquiries should be addressed to the corresponding authors.

## Abbreviations

The following abbreviations are used in this manuscript:

BAL	Bronchoalveolar lavage
CBC	Complete Blood Count
CT	Computed Tomography
LB2	Left caudal bronchus
LL	Latero-lateral
MRI	Magnetic Resonance Imaging
PCD	Primary Ciliary Dyskinesia
PCR	Polymerase Chain Reaction
TEM	Transmission Electron Microscopy
SCD	Secondary Ciliary Dyskinesia
VD	Ventro-dorsal
